# Combining ZooMS and zooarchaeology to study Late Pleistocene hominin behaviour at Fumane (Italy)

**DOI:** 10.1038/s41598-019-48706-z

**Published:** 2019-08-26

**Authors:** Virginie Sinet-Mathiot, Geoff M. Smith, Matteo Romandini, Arndt Wilcke, Marco Peresani, Jean-Jacques Hublin, Frido Welker

**Affiliations:** 1 Department of Human Evolution, Max Planck Institute for Evolutionary Anthropology, Leipzig, Germany; 2 University of Bologna, Department of Cultural Heritage, Ravenna, Italy; 3 University of Ferrara, Department of Humanities, Section of Prehistory and Anthropology, Ferrara, Italy; 4 Fraunhofer Institute for Cell Therapy and Immunology, Leipzig, Germany; 5 Section for Evolutionary Genomics, the Globe Institute, University of Copenhagen, Copenhagen, Denmark

**Keywords:** Biological techniques, Anthropology, Archaeology, Palaeontology

## Abstract

Collagen type I fingerprinting (ZooMS) has recently been used to provide either palaeoenvironmental data or to identify additional hominin specimens in Pleistocene contexts, where faunal assemblages are normally highly fragmented. However, its potential to elucidate hominin subsistence behaviour has been unexplored. Here, ZooMS and zooarchaeology have been employed in a complementary approach to investigate bone assemblages from Final Mousterian and Uluzzian contexts at Fumane cave (Italy). Both approaches produced analogous species composition, but differ significantly in species abundance, particularly highlighted by a six fold-increase in the quantity of *Bos/Bison* remains in the molecularly identified component. Traditional zooarchaeological methods would therefore underestimate the proportion of *Bos*/*Bison* in these levels to a considerable extent. We suggest that this difference is potentially due to percussion-based carcass fragmentation of large *Bos/Bison* bone diaphyses. Finally, our data demonstrates high variability in species assignment to body size classes based on bone cortical thickness and fragment size. Thus, combining biomolecular and traditional zooarchaeological methods allows us to refine our understanding of bone assemblage composition associated with hominin occupation at Fumane.

## Introduction

Zooarchaeological analyses use faunal remains to address archaeological questions. This provides a wealth of information on local and regional palaeoenvironments, the timing of hominin occupation, and interactions with other species^1–5^. Most specifically, such studies have been used to reconstruct hominin diet and subsistence patterns. However, faunal remains are often highly fragmented by taphonomic, including anthropogenic processes, precluding any type of taxonomic identification for most specimens. The non-identifiable component of Pleistocene bone assemblages frequently incorporates 60–70% of the excavated assemblage^6,7^. This leads to an extensive taxonomically uninformative proportion of bone assemblages, which could represent a source of bias in zooarchaeological studies of hominin subsistence behaviour.

Bone fragmentation can also provide a wealth of detail about site formation and depositional processes, but also more specifically about butchery practices and subsistence patterns. The species body part representation and the occurrence and location of cut-marks, percussion traces and bone breakage patterns can illustrate specific transport decisions by human groups^8–10^. However, large portions of bone assemblages remain taxonomically unidentifiable, and in the best cases can only be attributed to body size classes. Patterns of human subsistence behaviour are therefore often reliant on a relatively small proportion of morphologically identifiable remains. To provide a more comprehensive picture of human subsistence behaviour at a site requires the synthesis and analysis of comparable taxonomic and taphonomic data from both identifiable and unidentifiable fraction of Pleistocene faunal assemblages.

With the advancement of biomolecular studies in the past 20 years, different methods have been developed in order to aid the identification and the analysis of biological markers preserved in unidentifiable bone fragments. First, ancient DNA metabarcoding of bone samples has been employed to study the taxonomic composition of hundreds or thousands of bone samples simultaneously^11–13^. Second, various approaches involving ancient DNA sequencing have allowed the identification of vertebrate DNA directly from Pleistocene soil and sediment samples^14–17^. Both approaches provide qualitative insights into species composition but,currently, little resolution in terms of quantitative aspects^11^. In addition, all genetic and genomic approaches rely on ancient DNA survival, a biomolecule prone to fragmentation in comparison to other biomolecules, such as proteins^18–20^. Therefore, proteomic approaches, in particular collagen type I peptide mass fingerprinting through Zooarchaeology by Mass Spectrometry analysis (ZooMS^21^), have been suggested as a biomolecular alternative to study the taxonomic composition of the unidentifiable component of Pleistocene bone assemblages. Proteins such as collagen type I are phylogenetically informative, easily accessible, and survive beyond the temporal range of ancient DNA^22–24^.

ZooMS is a proteomic approach that allows taxonomic identification based on protein amino acid sequence variation through peptide mass fingerprinting^21^. This method is commonly performed on individual bone specimens in a targeted manner (for example on bone tools, particular taxonomic groups, or for radiocarbon or isotopic studies^25–28^) and thereby provides quantitative datasets potentially comparable with traditional zooarchaeological studies. ZooMS can add additional information on hominin behaviour in relation to faunal carcass processing and selection^29,30^, but this potential has not been explored. Nevertheless, previous studies have demonstrated that ZooMS is a robust tool that provides a high identification success rate (>95%) in the European Late Pleistocene. Initial taxonomic identifications through ZooMS have allowed the recovery of additional hominin specimens^30–33^. Bone specimens individually identified through ZooMS can be utilised in subsequent ancient DNA, isotopic, and radiocarbon dating analysis^34–37^. Finally, peptide mass fingerprints of collagen type I provide specimen-specific information of molecular diagenesis, allowing insights into spatial and temporal biomolecular preservation within a site^38–40^.

**Figure 1:**
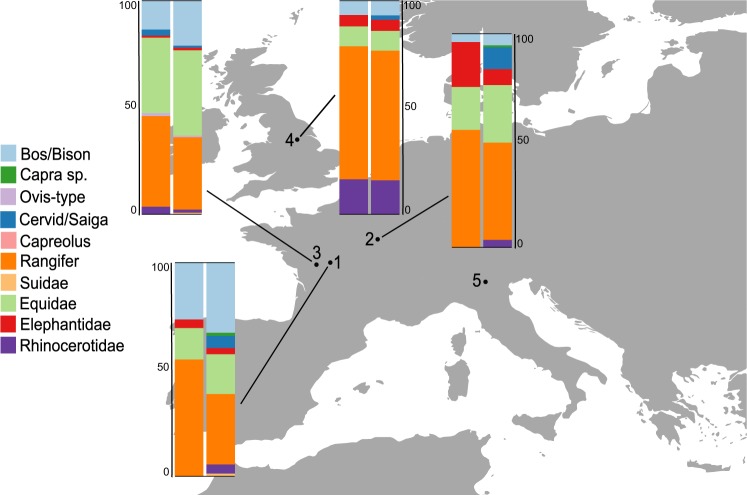
Site location of Fumane and other published, non-targeted ZooMS studies with zooarchaeological data available for the same archaeological layers. For each site the barplot indicates the percentage of number of identified specimens (%NISP) of herbivores for the morphologically identified (left) and the ZooMS-component (right). 1: Les Cottés (France)^29^ (ZooMS: N = 70, Morph: N = 75), 2: Grotte du Renne (France)^30^ (ZooMS: N = 108, Morph: N = 100), 3: Quinçay (France)^38^ (ZooMS: N = 412, Morph: N = 213), 4: Pin Hole Cave (UK)^41^ (ZooMS: N = 72, Morph: N = 78), 5: Fumane (Italy; this study see Fig. 3). Further details are provided in Supplementary Table S1.

In previous studies, ZooMS- and morphologically-identified components from the same layers are comparable in terms of species composition and abundance (Fig. 1). On some sites, the application of this method has allowed for the identification of species previously unconfirmed through traditional morphological analysis^29,30,38,41^ However, no ZooMS studies have investigated the relationship between faunal composition and bone fragmentation and, in turn, whether this is related to specific hominin behaviour at a site. In this study, 684 bone specimens across the Middle to Upper Palaeolithic transition (MUPT) corresponding to layers A6 to A2 at Fumane (Italy), have been analysed, with a focus on the Final Mousterian layer A4 (previously attributed to the Uluzzian: see ref.^42^) and the Uluzzian layer A3^43–46^.

## Methods

### Fumane

Fumane cave is located at the bottom of the Venetian Pre-Alps within the Western Monti Lessini in North of Italy (Fig. 1). The site has been known since the late 19th century, and was first excavated in 1964 by the Natural History Museum of Verona. The current excavations are led by a team from the University of Ferrara, and the faunal assemblage from these excavations were sampled and analysed to form the basis of this study.

The cave is part of a karst system composed of several cavities which has permitted the accumulation of a sedimentary sequence including Mousterian, Uluzzian and Aurignacian cultural complexes^45–49^. Human occupation at Fumane is attested by numerous faunal remains, lithics artefacts and combustion features. The site also offers unusual finds such as ornamental objects, painted stones, and evidence for the intentional removal of feathers from birds^50–53^. Various studies have presented radiocarbon dates, Uranium-Thorium dates, and electron spin resonance (ESR) combined dates, that provide a clear chronological framework for the entire stratigraphy^47,54^, in addition to palaeoecological contexts^55^. Within this framework, layers A4 and A3 date between 41.3 and 39.1 ka (Table 1)^56,57^.

**Table 1:** Fumane stratigraphy, chronological age, and faunal composition based on morphologically identifiable bone specimens.

Layer	Cultural attribution	Approximate age	Dominant faunal components (%NISP)
D3	Aurignacian		Ibex (*Capra ibex*, 43.0%)
D6	Aurignacian		Ibex (*Capra ibex*, 35.5%)
A1	Protoaurignacian		Ibex (*Capra ibex*, 43.9%), red deer (*Cervus elaphus*, 18.4%)
A2-A2R	Protoaurignacian	41–38 ka cal BP	Ibex (*Capra ibex*, 49.5%), red deer (*Cervus elaphus*, 18.8%)
A3	Uluzzian	44–42 ka cal BP	Red deer (*Cervus elaphus*, 29.5%), ibex (*Capra ibex*, 20.3%)
A4	Final Mousterian (Levallois)	44–42 ka cal BP	Red deer (*Cervus elaphus*, 39.3%), ibex (*Capra ibex*, 20.3%)
A5-A6	Mousterian (Levallois)	45–44 ka cal BP	Red deer (*Cervus elaphus*, 70.3%), roe deer (*Capreolus capreolus*, 11.7%)
A7	(-)		No human presence
A9	Mousterian (discoidal)	>47.6 ka cal BP	Red deer (*Cervus elaphus*, 39.3%), roe deer (*Capreolus capreolus*, 22.3%)
A10	Mousterian (Levallois/discoidal)	>47.6 ka cal BP	Roe deer (*Capreolus capreolus*, 43.8%), red deer (*Cervus elaphus*, 29.5%)
A11	Mousterian (Levallois)	>47.6 ka cal BP	Roe deer (*Capreolus capreolus*, 39.5%), red deer (*Cervus elaphus*, 32.3%)

Reference data on chronology taken from^44,47,56^. Reference data for zooarchaeological analysis taken from^43,51,58–60,115^. Note that layer A4 is now attributed to the Final Mousterian. See discussion in^42^.

The bone assemblages from Fumane are highly fragmented across the stratigraphy^43,58–60^. For example, for layers A3 and A4 about 3% of the assemblage (1,188 out of 36,944 bone remains including dental remains) can be securely identified based on morphological characteristics. For these 2 layers, the faunal spectrum based on the morphologically identifiable bones includes various ungulates, carnivores and birds, which together indicate a closed wooded environment indicative of temperate to cool climatic conditions^43,59^. The differences in faunal composition between layers A3 and A4 are relatively minor, and they occur in the abundance of the dominant species (Table 1).

### Zooarchaeological analysis

In the zooarchaeological analysis of the bone assemblages from Fumane, all the remains have been counted and grouped by size (0–1 cm, 1–2 cm, 2–3 cm, 3–4 cm, 4–5 cm, >5 cm). Burned and calcined bones were separated from the unburned specimens. All bone specimens were also grouped by body size class (large, medium-large, medium, medium-small, and small) based on bone cortical thickness and fragment size.

Taxonomic and skeletal identification was based on two reference collections. The first is stored at Lazio Museum Pole at the National Prehistoric Ethnographic Museum “Luigi Pigorini” in the Bioarchaeology Section in Rome, while the second is in the Prehistoric and Anthropological Sciences Section at the University of Ferrara. Microscopic analyses of the bone surfaces were carried out using portable low-magnification lenses (10–20X) and Leica S6D Green Ough stereomicroscopes with 0.75–70X magnification range. In specific cases, observation was also carried out using scanning electron microscopy (SEM).

In order to determine the nature of surface bone alterations, and to distinguish hominin from animal traces, trampling abrasion, and modern mechanical modifications produced by excavation tools, reference was made to the well-established taphonomic literature^61–68^. The degree of combustion was evaluated employing the methodology developed by Stiner *et al*.^69^. All faunal specimens were analysed, regardless of their taxonomic identifiability by one of the authors (M.R.) using traditional morphological observation. For our study, species abundance was assessed using the number of identified specimens (NISP)^70^, as minimum number of skeletal element (MNE) and minimum number of individuals (MNI) cannot be compared quantitatively with ZooMS data, which is inherently a NISP count. The percentage of the number of identified specimens have been calculated based on the taxonomically identified faunal specimens. Finally, bone fragmentation indices were calculated to evaluate the skeletal representation of the different animals and the skeletal survival rate^61,62,67^.

### ZooMS

684 morphologically unidentifiable bone and dental (dentine) specimens have been randomly sampled across levels A6 to A2 excavated in the same squares in the western area of the cave entrance (Supplementary Table S2). The majority of these bone specimens (73%) derive from the two layers A3 and A4. All selected specimens were recorded as individual specimens during excavation. For bone specimens, selection was based on the presence of cortical bone surface and a length of at least 2 cm. Dental specimens comprise a minor proportion of the analyzed samples (n = 8, 1.2%) and were excluded from surface modification analysis. For layers A3 and A4, our sampling covered the same spatial distribution (Supplementary Table S2). The maximum length of the bone specimens was measured individually with a digital calliper. ZooMS-identified bone specimens had previously been analysed morphologically and various taphonomic attributes recorded, allowing for the comparison of surface modification frequencies related to taphonomic and anthropogenic processes present in both components of the bone assemblages.

ZooMS extraction methods followed protocols outlined in detail elsewhere^30^. In short, soluble collagen is first extracted from small bone samples (<20 mg) by incubation in 100 µL 0.6 M ammonium-bicarbonate buffer at 65 °C for 1 hour. Half of this is digested using trypsin (0.5 μg/μL, Promega) overnight, acidified to pH < 1 using TFA (10% TFA), and cleaned on C18 ZipTips (Thermo Scientific). Hereafter, this is referred to as the “AmBic” extraction method^40^. Digested peptides are subsequently spotted in triplicate on a MALDI Bruker plate (MTP AnchorChip 384) with the addition of α-Cyano-4-hydroxycinnamic acid (CHCA) matrix. MALDI-TOF MS analysis was conducted at the University of York on an Ultraflex mass spectrometer (Bruker) in the mass-to-charge range 900–4000 m/z. MALDI-TOF stands for Matrix-assisted Laser-Desorption/Ionization, a method to ionize molecules, and is based on the co-crystallization of the matrix and an analyte, i.e. the substance to be analysed, in this case a bone proteome digested with trypsin. Analyte molecules are incorporated into the matrix while crystallization takes place. Subsequent laser impulses result in the detachment of crystalline particles into the vacuum of the mass spectrometer. Based on their time-of-flight (TOF) to the spectrometer´s detector, the molecular mass(es) of the analyte can be determined. Triplicates were merged for each sample, and taxonomic identification proceeded through peptide marker mass identification in comparison to a published database containing peptide marker series for all medium-to larger sized mammalian genera in existence in Europe during the Pleistocene^21,30^.

For 24 samples, the AmBic taxonomic identity based on soluble collagen was verified through subsequent demineralization of the sample in 0.6 M HCl, neutralization to pH 6–7, and protein solubilization again in 0.6 M ammonium-bicarbonate (hereafter the “HCl” extraction method)^21^. All subsequent steps for these 24 specimens were identical to the “AmBic” extraction method except that they were analysed at the MALDI-TOF MS facility at the Fraunhofer IZI in Leipzig, Germany, using an autoflex speed LRF MALDI-TOF (Bruker) in reflector mode, positive polarity, matrix suppression of 590 Da, and collected in the mass-to-charge range 800–4000 m/z.

Soluble collagen deamidation was calculated for selected peptides frequently observed in peptide fingerprints of collagen type I through published protocols^71,72^. Glutamine deamidation has been suggested as an indicator of collagen preservation variability^38,72^. Only slow-deamidating peptides have been observed to be frequently present in the Fumane spectra, and we hence limit our analyses to these peptides (P1105 and P1706). Deamidation ratios are presented on a scale from 0 (complete deamidation, all glutamines converted into glutamic acid) to 1 (no deamidation, all glutamines unmodified).

All analyses were conducted in R^73^, and figures were produced using the package ggplot2^74^.

## Results

Our analysis resulted in successful ZooMS identifications for 97.8% of a total of 684 bone specimens, with nearly identical success rates across all sampled levels (Supplementary Table S2). Deamidation values for all bone specimens indicate a temporal cline towards more extensive diagenetic modification for older layers (Supplementary Fig. S1). Extraction blanks to monitor protein contamination in the lab were empty of collagen type I. Furthermore, HCl demineralization and MALDI-TOF-MS analysis of a randomly selected set of 24 *Bos/Bison* specimens after AmBic analysis resulted in identical taxonomic identifications for both AmBic and HCl extraction methods (Supplementary Fig. S2). Our results are therefore difficult to explain by (laboratory) protein contamination.

Species presence in A3 and A4 is consistent between the ZooMS- and morphology-components of both levels (Supplementary Table S3). Exceptions are the addition of *Elephantidae* and *Rhinocerotidae* through ZooMS analysis for layer A4 and the presence of several carnivore species in the morphology-component^43^. This observation is similar to those made for previous untargeted ZooMS studies^29,30,38^. There are no herbivore species identified morphologically that are not represented in the ZooMS-component.

**Figure 2:**
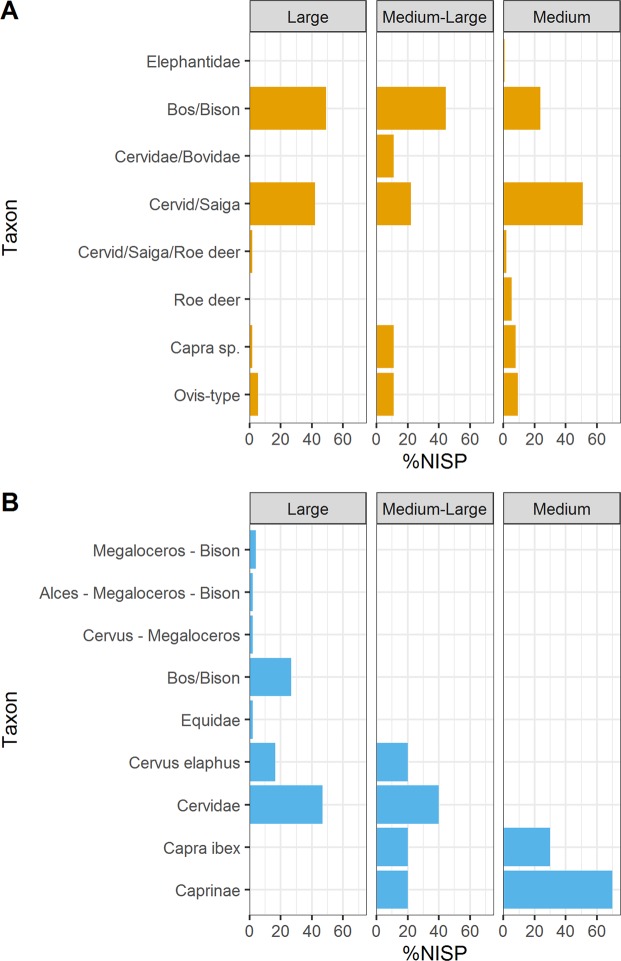
Barplot illustrating relative frequency (%NISP) for taxa identified using ZooMS (**A**) and morphology (**B**) in relation to their body size class attribution.

In zooarchaeology, bone specimens are frequently categorised in body size classes when species identification is not possible based on morphological criteria. At Fumane, bone fragment size and cortical thickness has been used as a proxy for body size class assignments. ZooMS analysis of bone specimens with body size class (BSC) assignments reveals that such categorizations are highly variable. For example, we note the presence of *Caprinae* within the large body size class and bone fragments identified as *Elephantidae* and *Bos/Bison* assigned to the medium body size class (Fig. 2). Thus, using the Fumane dataset, we illustrate that attributing taxonomically unidentifiable components to body size class categories (large, medium-large, medium) remains a useful, but problematic, qualitative tool. Moreover, these attributions are not taxonomically reliable as bone fragment size and cortical thickness are dependent on numerous overlapping and interrelated biological and taphonomic factors. As such, these body size class categories may not accurately reflect overall species composition at a site.

**Figure 3:**
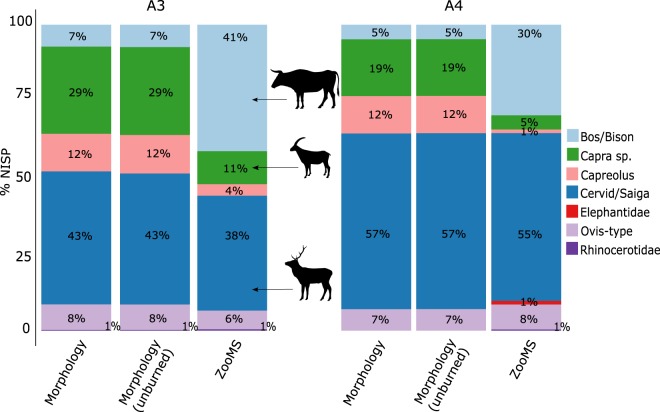
Barplot of %NISP of identified herbivores at Fumane. Morphology: this includes all specimens identified morphologically. Morphology (unburned): this includes specimens identified morphologically but excludes burned fragments (A3: 0.11% of burned specimens out of the morphology-identified assemblage (N = 453); (A4) 0.16% of burned bone fragments out of the morphological faunal assemblage (N = 681)). ZooMS: all specimens identified through ZooMS analysis (does not include burned fragments; see text for details). Colours are similar to Fig. 1. Data for the morphology-component derives from Tagliacozzo *et al*.^43^. Animal silhouettes are not to scale and derive from phylopic.org.

In contrast to previous studies (Fig. 1), a large difference in the quantitative composition of the ZooMS-component and the morphologically-identified component have been observed for layers A3 and A4 (Fig. 3). As ZooMS analysis cannot be performed on burned bone it was important to have comparable datasets for both the morphological and ZooMS component. Therefore, we assessed the proportion of burned and unburned specimens by taxon in the morphological component from A3 and A4 (Fig. 3). The species representation is similar for both burned and unburned portions.

Overall, species representation among layers A3 and A4 is driven by an almost 6-fold increase in the number of *Bos/Bison* specimens in the ZooMS-component (36%) compared to the morphology-component (6%) (Fig. 3), and counterbalanced by a relative decrease in the number of specimens attributed to *Capra* sp. Such a frequency difference in the presence of a particular species between the ZooMS- and morphology-components of the same archaeological layer has never been observed until now (Fig. 1). The remainder of this paper aims to explore potential causes of this compositional difference by focusing on the three main taxonomic components (*Capra* sp., Cervid/Saiga, and *Bos/Bison*) of the layers A3 and A4.

**Figure 4:**
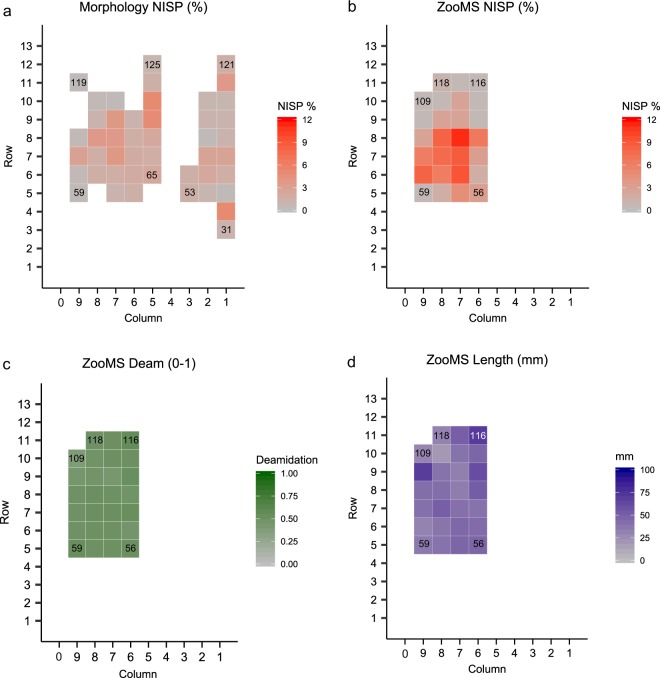
Spatial distribution maps of all bone specimens from the species groups Cervid/Saiga, *Capra* sp. and *Bos/Bison* from layers A3 and A4 at Fumane cave. (**a**) Distribution of %NISP of the three identified species for the morphology-component, over the sampled squares. (**b**) Distribution of %NISP of the three identified species for the ZooMS-component, over the sampled squares. (**c**) Average deamidation per square for the ZooMS component. (**d**) Average length (mm) per square for the ZooMS component. Squares are 1 × 1 meter, and the corresponding excavation numbers for each square can be obtained by joining the y-axis number and the x-axis number (for a detailed excavation plan, see^59^). The numbers within the squares represent the square numbers from the excavation grid. For **a** and **b**, a %NISP of 12% would indicate that 12% of the NISP of the combined total of *Capra* sp., *Bos*/*Bison*, and Cervid/Saiga is derived from that square.

**Figure 5:**
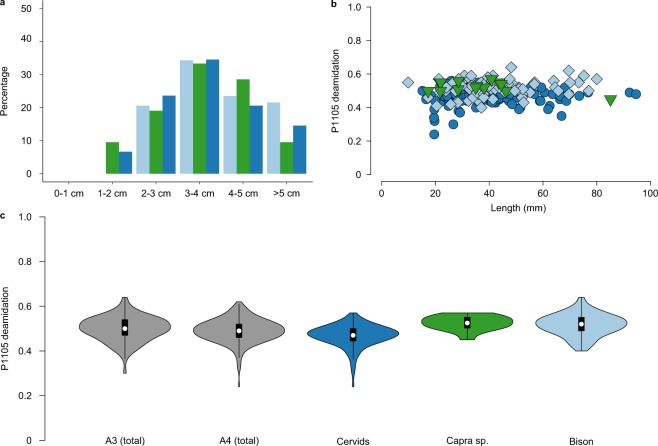
Taphonomic and molecular preservation of Cervid/Saiga, *Capra* sp., and *Bos/Bison* specimens. (**a)** Bone length distribution in mm. (**b)** Absence of a relationship between bone length (mm) and molecular diagenesis (P1105 deamidation). (**c**) Violin plots of P1105 deamidation. A3 and A4 include all specimens identified through ZooMS for these levels. Note that Cervid/Saiga is the dominant species group for both A3 and A4, significantly influencing the total violin plots for both levels displayed on the left. Only data for A3 and A4 are included for each panel. Colour legend is identical across panels as well as Figs. 1 and 3 (Cervid/Saiga: dark blue, *Capra* sp.: green and *Bos/Bison*: light blue).

For these three species groups, the spatial distribution of the bone specimens is more restricted in the ZooMS component (Fig. 4). The studied bone fragments have nearly identical distributions of specimen length (Fig. 5a). Whilst *Bos/Bison* specimens (41.7 ± 16.9 mm) are longer than *Capra* sp. specimens (37.1 ± 16.0 mm) and Cervid/Saiga specimens (39.6 ± 15.0 mm), there is no significant difference in the overall distributions (Cervid/Saiga versus *Capra* sp.: t-test(0.7), df = 20, *p* = 0.48; Cervid/Saiga versus *Bos/Bison*: t-test(−1.1), df = 178, *p* = 0.27; *Capra* sp. versus *Bos/Bison*: t-test(−1.2), df = 22, *p* = 0.23). However, considering that bone specimens of over 2 cm in length have been selected for this study, the distribution might not be similar for the smallest, unstudied, size range (0–2 cm). Finally, there is no apparent difference in the spatial distribution of bone fragment size (Fig. 4d). Altogether, we therefore conclude that, assuming *Bos*/*Bison* individuals are generally larger than *Capra* sp. and Cervid/Saiga individuals, *Bos*/*Bison* bone elements have been subjected to a larger amount of fragmentation.

Bone length and P1105 deamidation, an indicator of molecular collagen type I preservation, display no significant relationship for any of the three species groups (Fig. 5b; Spearman rank correlation, R_s_ = 0.09 *p* = 0.19). All specimens from layers A3 and A4 display an identical distribution of P1105 deamidation (t-test(1.5), df = 420, *p* = 0.14) (Fig. 5c) and show no spatial differences in the amount of average deamidation per square in the area analyzed (Fig. 4c). However, Cervid/Saiga specimens have a deamidation distribution significantly different from that observed for *Capra* sp. and *Bos/Bison* specimens (Cervid/Saiga versus *Capra* sp.: t-test(−5.9), df = 21, *p* = 6.4*10^−06^; Cervid/Saiga versus *Bos/Bison*: t-test(−6.8), df = 171, *p* = 1.4*10^−10^), while *Capra* sp. and *Bos/Bison* specimens have similar distributions (t-test(0.9), df = 25, *p* = 0.39). This reveals that Cervid/Saiga specimens have undergone a different extent of molecular diagenesis, but not fragmentation, compared to bone specimens from the other ZooMS-identified species.

The frequency of bone surface modifications due to non-anthropogenic taphonomic processes (e.g., weathering, concretion, corrosion, mineral staining and root etching) is broadly similar for all three species groups in both the ZooMS and morphology component, as is the presence of carnivore and/or rodent marks (Supplementary Tables S4, S5). Thus, non-anthropogenic bone surface modifications appear to have affected all three species groups to a similar extent. Similarly, increased levels of molecular damage for Cervid/Saiga specimens can only be explained by a mechanism unrelated to bone fragmentation processes, but this cannot explain the increase in *Bos/Bison* specimens in the ZooMS component.

**Figure 6:**
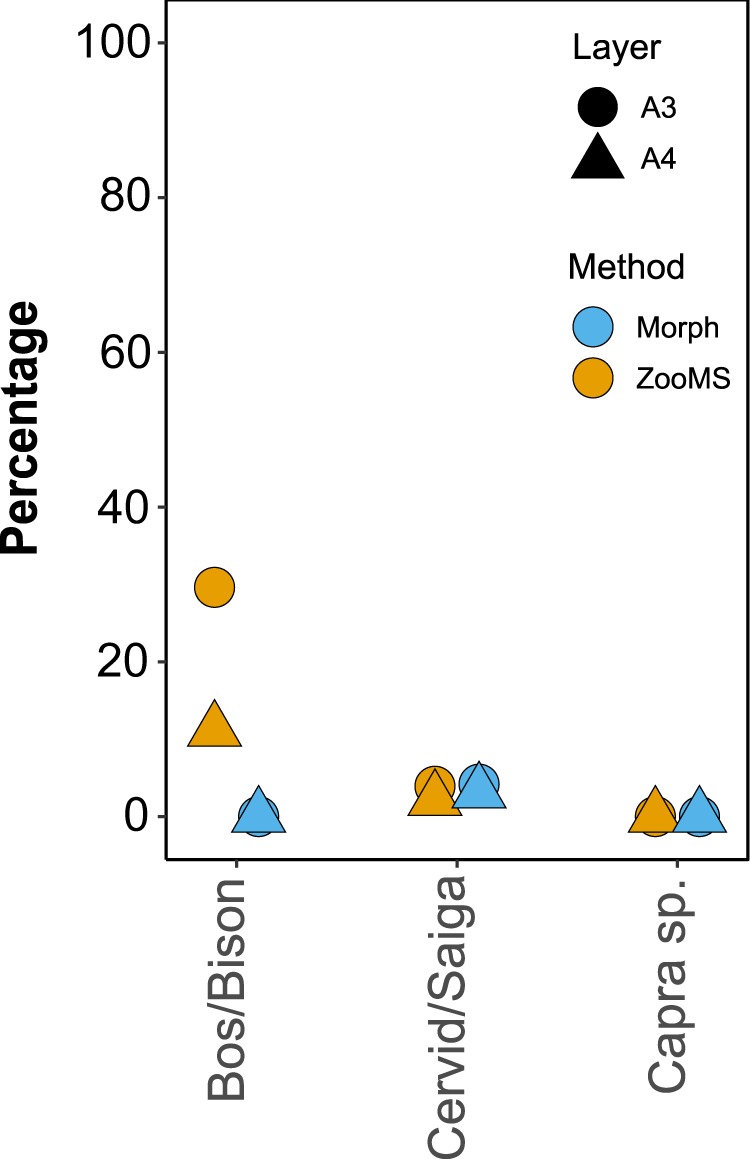
Percussion marks frequencies for the three main species groups within the morphology and the ZooMS-identified component in layers A3 and A4. Y-axis gives the percentage of occurrence (0–100) of percussion marks per specimen for the three major species groups (x-axis). Different point shapes indicate different layers (circle: A3; triangle: A4) whilst colour illustrates different identification method (blue: morphologically identified; gold: ZooMS). See Supplementary Table S4 for associated NISP numbers.

Likewise, bone surface modifications resulting from anthropogenic processes are present to a similar extent in the ZooMS- and morphologically-identified components. In general, such bone surface modifications are recorded in comparable frequencies, but the frequencies for cut marks and impact points (or loading point)^67^ are more distinct (Supplementary Table S4). It should be noted that frequencies differ between species to some extent, but generally not between the morphology- and ZooMS-components within the same species. For example, there seem to be fewer anthropogenic modifications of *Capra sp*. specimens compared to both Cervid/Saiga and *Bos/Bison* specimens (Supplementary Table S4). We note, however, high frequencies of percussion marks for *Bos/Bison* specimens in the ZooMS-component of both A3 (30%) and A4 (11%; Supplementary Table S4 and Fig. 6). Such marks are absent for *Bos/Bison* in the same layers (0% and 0%, respectively) in the morphology-component, mostly represented by bone epiphysis, but also by carpals, tarsals, and distal limb bones. This is in contrast to the ZooMS assemblages, which are mainly composed of long bone fragments (diaphysis) and ribs. Indeed, percussion marks on *Bos/Bison* specimens are exclusively present on long bone diaphyses in our ZooMS-identified sample set. These are bone elements subjected to more intense processing during butchering and bone marrow extraction^4,8,75^. Moreover, these specific traces appear to occur at much lower frequencies in the Cervid/Saiga (2–4%) and *Capra* sp. (0–0%) specimen groups.

All these observations related to bone surface modifications are replicated when the analysed specimens are restricted to the same set of squares for both the ZooMS and morphology components of the faunal assemblage (Supplementary Table S2). Alongside the absence of spatial patterns in bone fragmentation (Fig. 4d) and molecular degradation (Fig. 4c), there is therefore also no apparent spatial patterning in occurrence and frequency of bone surface modifications.

## Discussion

Palaeoproteomics, including ZooMS, is a recent addition to the molecular toolkit available to explore past faunal communities^21,29^, the phylogenetic relationships between those species^20,22,76,77^, and hominin interactions with their immediate environment^78^. ZooMS in particular has been adopted to survey the unidentifiable bone component of Palaeolithic sites in order to identify additional hominin remains^30–33^ and to explore the qualitative aspects of faunal assemblages. Archaeological complexes like the Uluzzian in Italy have been attributed to the transitional phase between the Middle and the Upper Palaeolithic marked by the diffusion of populations of anatomically and genetically modern hominins and the local extinction of Neanderthals^46,79–82^. However, few hominin remains are directly attributable to the Uluzzian. Those that are available are associated with complicated debates on their taphonomic history^83–85^, or cannot be reliably assigned to Neanderthals or modern hominins based on morphological characteristics because of their elevated degree of fragmentation or the uncertain stratigraphic position^86,87^. Although no additional hominin specimen attributable to the Uluzzian has been identified here, our dataset adds to a growing understanding of hominin interactions with the environment around the MUPT^88–92^.

Alongside similar methods based on ancient DNA sequencing, ZooMS has the ability to provide quantitative data on the abundance of particular species. This quantitative aspect has not been explored up to now, however, partly because previous studies indicated little quantitative difference between morphology-identified and ZooMS-identified components of the same assemblage (Fig. 1). Here we have encountered an assemblage where the morphology- and ZooMS-identified components are relatively similar in terms of species composition but markedly different in quantitative aspects for two distinct archaeological layers at the same site. In particular, a 6-fold increase in *Bos/Bison* specimens in the ZooMS-component. This is counterbalanced by a 3-fold decrease in *Capra* sp. (Fig. 3). We observe no apparent spatial differences in bone fragmentation (Fig. 4d) or molecular diagenesis (Fig. 4c). However, we note that Cervid specimens are more deamidated than other bone specimens at the site. It is therefore possible that the enhanced deamidation of Cervids in A3 and A4 is the result of hominin behaviour, although we are unable, at present, to precisely define which kind of anthropogenic process might be responsible. Possibilities include boiling, low-temperature roasting, or fermentation, but a precise assessment requires the development of further molecular methods to identify and distinguish these different anthropogenic processes. Furthermore, slightly higher rates of Cervid collagen deamidation cannot explain the higher incidence of *Bos/Bison* specimens.

Bones fractured deliberately to extract marrow have been previously noted in both layers at Fumane^43^. Compared to the morphologically identified assemblage, high frequencies of percussion marks on ZooMS-identified *Bos/Bison* specimens have been observed (Fig. 6). Therefore, the larger size of *Bos*/*Bison* elements and higher frequency of long bone diaphysis fragments and marrow fractures in the ZooMS-identified assemblage might explain the higher incidence of this species. Consequently, without the addition of the ZooMS dataset our interpretation of assemblage composition and human subsistence behaviour at Fumane would have been incomplete. The complementary ZooMS and zooarchaeological datasets from Fumane have provided a more comprehensive picture of assemblage composition and highlighted variation in the intensity and treatment of different prey sizes. This is exemplified by the increased fragmentation of *Bos/Bison* remains.

Palaeolithic faunal assemblages are often characterized by a high degree of fragmentation. This phenomenon can result from a number of natural taphonomic agents and processes^67,93–102^ but also due to intensive hominin carcass processing. Indeed, such patterns appear similar whether in the Lower^98,101,103–106^, Middle^2,107–111^ or Upper Palaeolithic^88,112^. Often, long bones and rib fragments represent, by far, the largest proportion of the unidentified component of faunal assemblages^109,113^. Similarly, these body regions represent high utility in terms of available resources (e.g., meat, marrow) and are thus frequently fragmented^6–8,114^. This, undoubtedly, leads to a loss of taxonomic identification and hominin behavioural information, with behavioural interpretations based on a relatively small proportion of identifiable remains. The novel application of ZooMS to taxonomically unidentifiable specimens has the potential to provide a clearer picture of overall species composition at a site and can help to reduce analyst error, especially when faced with a large proportion of one species within the morphologically identified component.

Comparisons of the relative proportions of species within the morphological and ZooMS components provides complementary data about species abundance and environmental context at sites though these datasets have not, to date, been used to address broader zooarchaeological questions related to site use, assemblage formation, or hominin subsistence behaviour. The current study presents a first attempt to integrate complementary data sets from zooarchaeological and ZooMS-based analyses. Whilst the morphologically-identified assemblage may be dominated by a small number of species, sometimes a single species, this may not necessarily reflect true assemblage abundance. Body size class based on bone cortical thickness can provide a qualitative assessment of assemblage fragmentation. Comparative analysis at Fumane illustrates considerable variation between the ZooMS and morphological datasets when assigning bone fragments to specific body size classes based on fragment size and cortical bone thickness. Subsequent ZooMS analysis illustrates a scattering of species across and within these categories (e.g. *Elephantidae* in the medium size class, *Capra* sp. in the large size class) (Fig. 2). Body size class attributions should therefore be used with caution. Instead, molecular approaches like ZooMS can provide a more secure assignment of taxonomic identity and gives a more informative picture of species proportions, and associated bone surface modifications, within an assemblage.

## Conclusion

Faunal remains from archaeological sites allow us to reconstruct how hominin populations adapted to changing climates and environments through the detailed study of patterns of hominin subsistence. Faunal analysis provides ecological information and also illustrates hominin behaviour associated with prey choice and carcass exploitation. High bone fragmentation rates, due to both natural and anthropogenic processes, result in low proportions of morphologically identifiable remains for many Palaeolithic faunal assemblages. Previous studies have relied solely on morphologically identifiable fauna, which can potentially exclude vast quantities of specimens and archaeologically valuable data. Through the biomolecular analysis of a large number of unidentifiable bone fragments, we have observed a significant quantitative difference in the ZooMS faunal spectrum compared to the morphologically identifiable portion within the same assemblage. This is most evident as a 6-fold increase in the number of *Bos/Bison* specimens in the morphologically unidentifiable fragments; this is possibly due to the size of *Bos*/*Bison* bone elements, their processing during food procurement, and differences in bone elements identified through molecular and morphological methods of taxonomic identification. We also demonstrate that assigning bone fragments to body size classes based on bone cortical thickness and fragment size is an unreliable predictor of taxonomic identity, and these categorizations should therefore be used cautiously in behavioural interpretations of assemblage formation. We have thereby demonstrated that combining molecular and traditional zooarchaeological analysis can provide additional complementary insights into Pleistocene faunal assemblages and hominin subsistence behaviour.

## Supplementary information

Supplementary Information
