# Supplementary material for: Combining ZooMS and zooarchaeology to study Late Pleistocene hominin behaviour at Fumane (Italy)
Source: Sci Rep. 2019 Aug 26;9:12350. doi: 10.1038/s41598-019-48706-z (PMC6710433; doi:10.1038/s41598-019-48706-z)
Supplement: Supplementary file 1 — Supplementary Information [file 41598_2019_48706_MOESM1_ESM.pdf]

# SUPPLEMENTARY INFORMATION TO:

# Combining ZooMS and zooarchaeology to study Late Pleistocene hominin behaviour at Fumane (Italy).

Virginie Sinet-Mathiot[1,*], Geoff M. Smith[1], Matteo Romandini[2,3], Arndt Wilcke[4], Marco Peresani[3,*], Jean-Jacques Hublin[1], Frido Welker[1,5,*]

[1] Department of Human Evolution, Max Planck Institute for Evolutionary Anthropology, Leipzig, Germany.
[2] University of Bologna, Department of Cultural Heritage, Ravenna, Italy.
[3] University of Ferrara, Department of Humanities, Section of Prehistory and Anthropology, Ferrara, Italy.
[4] Fraunhofer Institute for Cell Therapy and Immunology, Leipzig, Germany
[5] Section for Evolutionary Genomics, the Globe Institute, University of Copenhagen, Copenhagen, Denmark.

*Corresponding authors: Virginie Sinet-Mathiot (virginie_sinet@eva.mpg.de), Marco Peresani (psm@unife.it), Frido Welker (frido_welker@eva.mpg.de).

# SUPPLEMENTARY INFORMATION

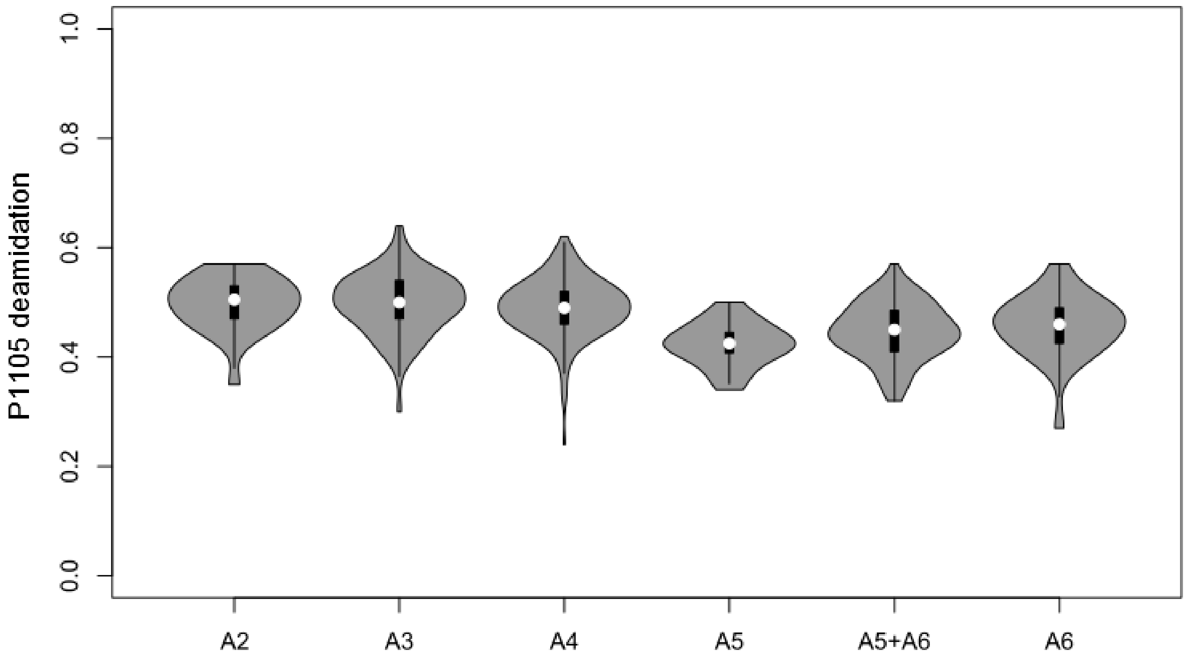


**Supplementary Figure S1. Temporal cline of bone P1105 deamidation values across the Fumane stratigraphy.** Chronologically younger layers (from A2) are less deamidated than chronologically older layers (to A6). P1105 deamidation distributions between different archaeological layers are significantly different at $p<0.05$ for all comparisons except between the layers A3 (Uluzzian) and A4 (Final Mousterian; see main text).

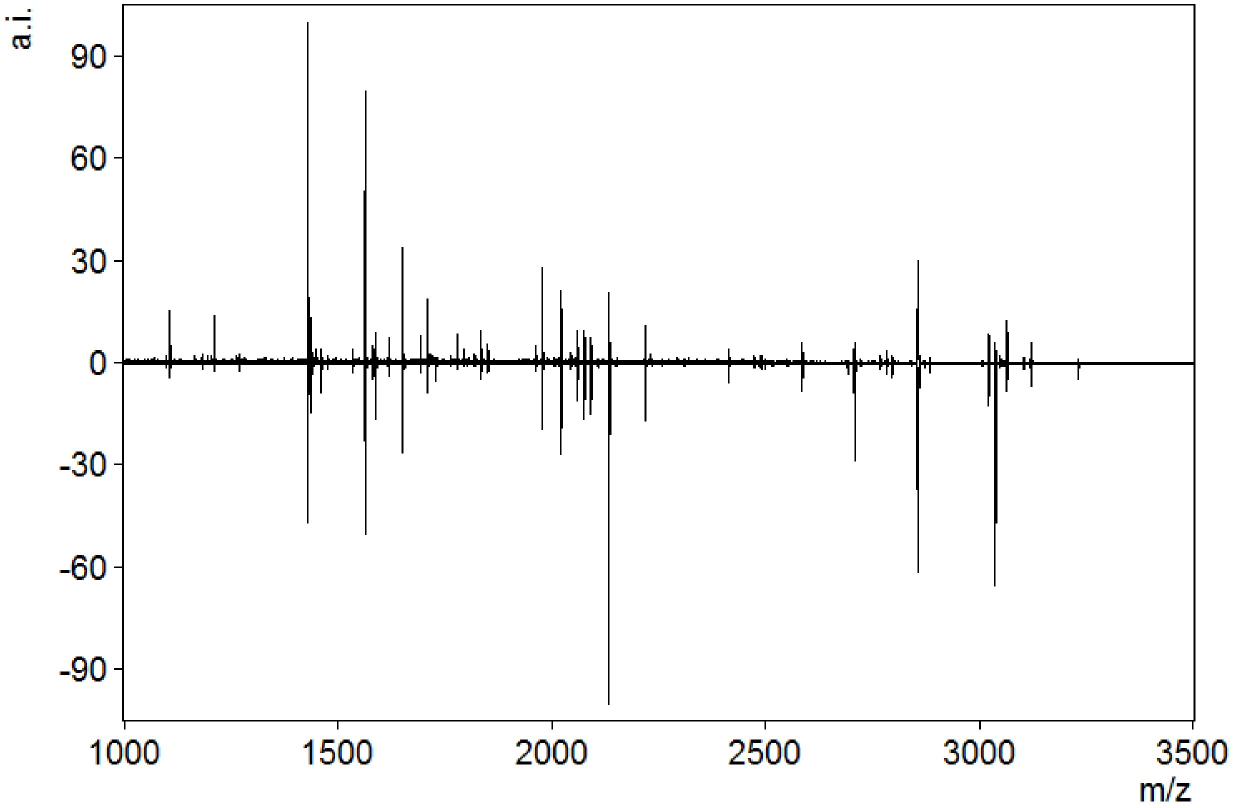


**Supplementary Figure S2. Comparison of an AmBic (top) and Acid (bottom) extract analysed in York and Leipzig, respectively.** Spectra are displayed in the m/z range of 1000-3500, and concern the same morphologically unidentifiable bone specimen (F-258; Bos/Bison). Y-axis (top) and inverted y-axis (bottom) shows relative intensity to the highest peak in either spectrum.

**Supplementary Table S1. Percentage of number of identified specimens (%NISP) of herbivores for the morphologically identified (Morph.) and the ZooMS-component (ZooMS) from Les Cottés (France), Quinçay (France), Pin Hole Cave (UK), and Grotte du Renne (France)**. Note that the values for Pin Hole are estimates as the original publication does not provide numeric information, only graphical.

| Species | Les Cottés ZooMS | Les Cottés Morph . | Quincay ZooMS | Quincay Morph. | Pin Hole ZooMS | Pin Hole Morph. | Grotte du Renne ZooMS | Grotte du Renne Morph. |
|---|---|---|---|---|---|---|---|---|
| *Bos/Bison* | 32.9 | 26.7 | 21.1 | 13.6 | 7.0 | 6.8 | 5.6 | 4.0 |
| *Capra* sp. | 1.4 | 0 | 0 | 0 | 0 | 0 | 0.9 | 0 |
| Ovis-type | 0 | 0 | 0.7 | 1.4 | 0 | 0 | 0 | 0 |
| Cervid/Saiga | 5.7 | 0 | 1.0 | 2.8 | 2.1 | 0 | 10.2 | 0 |
| *Capreolus capreolus* | 0 | 0 | 0 | 0 | 0 | 0 | 0 | 0 |
| *Rangifer tarandus* | 32.9 | 54.7 | 33.7 | 42.2 | 60.5 | 62.0 | 45.4 | 55.0 |
| Suidae | 1.4 | 0 | 1.0 | 0 | 0 | 0 | 0 | 0 |
| Equidae | 18.6 | 14.7 | 39.8 | 35.2 | 9.2 | 9.3 | 26.9 | 20.0 |
| Elephantidae | 2.9 | 4.0 | 1.2 | 0.9 | 5.2 | 5.3 | 7.4 | 21.0 |
| Rhinocerotidae | 4.3 | 0 | 1.5 | 3.8 | 16.0 | 16.5 | 3.7 | 0 |

**Supplementary Table S2. Squares, sublayers and layers studied with ZooMS.** For further information see Peresani et al. [44,81,92].

| Layers | Sublayers | Squares |
|---|---|---|
| A2 | A2R; A2+A2R | 116; 117; 127; 128 |
| A3 | A3; A3I; A3II; A3III; A3IV; A3-A4II; A3 tetto; | 56; 57; 58; 59; 66; 67; 68; 69; 76; 77; 78; 79; 86; 87; 88; 89; 97; 98 |
| A4 | A4; A4II; A4II/A5-A6; A4IV; A4V; A4V/A5-A6; A4V/A5; A4VI; A4VI/A5-A6; A4-A5 | 56; 57; 58; 59; 66; 67; 68; 69; 76; 77; 78; 79; 86; 87; 88; 89; 96; 97; 98; 99; 106; 107; 108; 109; 116; 117; 118 |
| A5 | A5 | 41; 53; 61; 62; 71; 72 |
| A5+A6 | A5+A6; A5+A6-A6 | 60; 65; 70; 75; 81; 90; 91; 95; 100; 101; 105; 106; 107; 108; 111; 116; 117; 118 |
| A6 | A6 | 51; 61; 80; 117; 118 |

**Supplementary Table S3. ZooMS species IDs for all studied levels.** Percentages in the bottom row indicate the identification success rate per level. In the context of Fumane, Cervid/Saiga can be attributed to either *Cervus elaphus* (red deer), *Megaloceros giganteus* (giant deer) or *Alces alces* (elk), as an attribution to *Saiga* sp. or *Dama* sp. can be excluded based on our knowledge of the fauna in this region for this period. Ovis-type includes non-*Capra* sp. members of the Caprinae. In the context of Fumane an attribution to *Rupicapra rupicapra* (chamois) is possible. Canidae includes members of *Canis* sp. and *Vulpes alopex* (arctic fox), both of which are known morphologically at Fumane, but based on the ZooMS marker series an attribution to *Vulpes vulpes* can be excluded. Felinae is represented morphologically by *Lynx lynx* at the site for the stratigraphic portion considered in this study, and is the most likely species candidate for this taxonomic group in the ZooMS component.

| **Species** | **A2** | **A3** | **A4** | **A5** | **A5+A6** | **A6** |
|---|---|---|---|---|---|---|
| Canidae (not red fox) | 0 | 1 | 4 | 0 | 0 | 0 |
| Red fox | 0 | 1 | 3 | 0 | 0 | 0 |
| Felinae | 0 | 2 | 1 | 0 | 0 | 0 |
| Ursidae | 0 | 0 | 7 | 0 | 5 | 1 |
| *Bos/Bison* | 7 | 88 | 76 | 1 | 7 | 1 |
| *Capra* sp. | 5 | 23 | 12 | 0 | 8 | 1 |
| Ovis-type | 1 | 13 | 21 | 2 | 3 | 1 |
| Cervid/Saiga | 11 | 80 | 141 | 15 | 86 | 22 |
| *Capreolus* sp. | 0 | 8 | 3 | 1 | 2 | 0 |
| Rhinocerotidae | 0 | 1 | 1 | 0 | 0 | 0 |
| Elephantidae | 0 | 0 | 3 | 0 | 0 | 0 |
| | | | | | | |
| Pantherinae/Hyaenidae | 0 | 1 | 0 | 0 | 0 | 0 |
| Cervidae/Bovidae | 0 | 1 | 0 | 0 | 0 | 0 |
| Cervid/Saiga/Roe deer | 0 | 3 | 3 | 1 | 0 | 0 |
| | | | | | | |
| Indeterminate | 0 | 0 | 2 | 0 | 4 | 0 |
| | | | | | | |
| **Total** | **24** (100%) | **222** (98%) | **277** (98%) | **20** (95%) | **115** (97%) | **26** (100%) |

**Supplementary Table S4. A3 and A4 surface modification frequencies for the three main species groups per identification method.** All agents of surface modification presented in the table below relate to the frequency of presence for each of these attributes. Data for all studied squares for both ZooMS and morphological analysis are included. Dental remains and burned specimens are not included in the calculation of the shown percentages. See Supplementary Table S5 for similar frequencies derived from a restricted set of squares. Numbers listed are percentages of occurrence and those in parentheses are number of specimens. ZooMS: bone component identified through ZooMS analysis. Morph.: bone component identified through morphology.

| | | **Cervid/Saiga** | | ***Capra* sp.** | | ***Bos/Bison*** | |
|---|---|---|---|---|---|---|---|
| | | **ZooMS** | **Morph.** | **ZooMS** | **Morph.** | **ZooMS** | **Morph.** |
| **Weathering** | A3 | 27.4 (14) | 15.6 (25) | 21.4 (3) | 15.6 (13) | 20.7 (11) | 40.0 (8) |
| | A4 | 43.0 (40) | 21.9 (40) | 25.0 (1) | 24.0 (12) | 15.5 (7) | 52.9 (9) |
| **Concretion** | A3 | 33.3 (17) | 26.8 (43) | 14.2 (2) | 32.5 (27) | 24.5 (13) | 60.0 (12) |
| | A4 | 20.4 (19) | 39.0 (71) | 50.0 (2) | 22.0 (11) | 17.7 (8) | 52.9 (9) |
| **Corrosion** | A3 | 15.6 (8) | 5.6 (9) | 42.8 (6) | 24.0 (20) | 20.7 (11) | 30.0 (6) |
| | A4 | 10.7 (10) | 9.9 (18) | 50.0 (2) | 18.0 (9) | 20.0 (9) | 17.6 (3) |
| **Exfoliation** | A3 | 5.9 (3) | 25.6 (41) | 0.0 (0) | 15.6 (13) | 16.9 (9) | 35.0 (7) |
| | A4 | 10.7 (10) | 22.5 (41) | 0.0 (0) | 18.0 (9) | 11.1 (5) | 23.5 (4) |
| **Mineral staining** | A3 | 29.4 (15) | 16.8 (27) | 21.4 (3) | 16.8 (14) | 9.4 (5) | 15.0 (3) |
| | A4 | 25.8 (24) | 32.4 (59) | 25.0 (1) | 34.0 (17) | 13.3 (6) | 5.9 (1) |
| **Root etching** | A3 | 64.7 (33) | 59.3 (95) | 50.0 (7) | 44.5 (37) | 49.0 (26) | 60.0 (12) |
| | A4 | 69.8 (65) | 53.2 (97) | 75.0 (3) | 46.0 (23) | 57.7 (26) | 47.0 (8) |
| **Carnivore and/or Rodent marks** | A3 | 5.9 (3) | 1.9 (3) | 14.2 (2) | 15.6 (13) | 1.9 (1) | 25.0 (5) |
| | A4 | 3.2 (3) | 3.8 (7) | 0.0 (0) | 4.0 (2) | 8.9 (4) | 0.0 (0) |
| **Cut marks** | A3 | 58.8 (30) | 36.8 (59) | 35.7 (5) | 24.0 (20) | 32.0 (17) | 50.0 (10) |
| | A4 | 44.0 (41) | 48.9 (89) | 25.0 (1) | 16.0 (8) | 13.3 (6) | 41.1 (7) |
| **Impact points** | A3 | 7.8 (4) | 21.8 (35) | 7.14 (1) | 4.81 (4) | 15.0 (8) | 10.0 (2) |
| | A4 | 13.9 (13) | 19.7 (36) | 0.0 (0) | 6.0 (3) | 24.4 (11) | 35.2 (6) |
| **Percussion Marks** | A3 | 3.9 (2) | 4.4 (7) | 0.0 (0) | 0.0 (0) | 30.1 (16) | 0.0 (0) |
| | A4 | 2.2 (2) | 3.3 (6) | 0.0 (0) | 0.0 (0) | 11.1 (5) | 0.0 (0) |
| **Total NISP** | A3 | 51 | 160 | 14 | 83 | 53 | 20 |
| | A4 | 93 | 182 | 4 | 50 | 45 | 17 |

**Supplementary Table S5. A3 and A4 surface modification frequencies for the three main species groups per identification method, derived from the squares also covered by ZooMS sampling.** All agents of surface modification presented in the table below relate to the frequency of presence for each of these attributes. Data for all studied squares for both ZooMS and morphological analysis are included. Dental remains and burned specimens are not included in the calculation of the shown percentages. Numbers listed are percentages of occurrence and those in parentheses are number of specimens. ZooMS: bone component identified through ZooMS analysis. Morph.: bone component identified through morphology.

| | | **Cervid/Saiga** | | ***Capra* sp.** | | ***Bos*/*Bison*** | |
|---|---|---|---|---|---|---|---|
| | | **ZooMS** | **Morph.** | **ZooMS** | **Morph.** | **ZooMS** | **Morph.** |
| **Weathering** | A3 | 27.4 (14) | 35.0 (7) | 21.4 (3) | 23.0 (3) | 20.7 (11) | 50.0 (3) |
| | A4 | 43.0 (40) | 37.2 (19) | 25.0 (1) | 63.6 (7) | 15.5 (7) | 85.7 (6) |
| **Concretion** | A3 | 33.3 (17) | 15.0 (3) | 14.2 (2) | 15.3 (2) | 24.5 (13) | 50.0 (3) |
| | A4 | 20.4 (19) | 21.5 (11) | 50.0 (2) | 9.1 (1) | 17.7 (8) | 57.1 (4) |
| **Corrosion** | A3 | 15.6 (8) | 20.0 (4) | 42.8 (6) | 38.4 (5) | 20.7 (11) | 33.3 (2) |
| | A4 | 10.7 (10) | 11.7 (6) | 50.0 (2) | 27.2 (3) | 20.0 (9) | 14.2 (1) |
| **Exfoliation** | A3 | 5.88 (3) | 15.0 (3) | 0.0 (0) | 7.7 (1) | 16.9 (9) | 16.6 (1) |
| | A4 | 10.7 (10) | 25.4 (13) | 0.0 (0) | 9.1 (1) | 11.1 (5) | 28.5 (2) |
| **Mineral staining** | A3 | 29.4 (15) | 15.0 (3) | 21.4 (3) | 7.7 (1) | 9.4 (5) | 0.0 (0) |
| | A4 | 25.8 (24) | 47.0 (24) | 25.0 (1) | 27.2 (3) | 13.3 (6) | 0.0 (0) |
| **Root etching** | A3 | 64.7 (33) | 65 (13) | 50.0 (7) | 61.5 (8) | 49.0 (26) | 50.0 (3) |
| | A4 | 69.8 (65) | 43.1 (22) | 75.0 (3) | 45.4 (5) | 57.7 (26) | 57.1 (4) |
| **Carnivore and/or rodent marks** | A3 | 5.88 (3) | 10.0 (2) | 14.2 (2) | 15.3 (2) | 1.88 (1) | 16.6 (1) |
| | A4 | 3.22 (3) | 1.96 (1) | 0.0 (0) | 0.0 (0) | 8.9 (4) | 0.0 (0) |
| **Cut marks** | A3 | 58.8 (30) | 55.0 (11) | 35.7 (5) | 15.3 (2) | 32.0 (17) | 33.3 (2) |
| | A4 | 44.0 (41) | 52.9 (27) | 25.0 (1) | 18.1 (2) | 13.3 (6) | 71.4 (5) |
| **Impact points** | A3 | 7.8 (4) | 15.0 (3) | 7.1 (1) | 7.7 (1) | 15.0 (8) | 33.3 (2) |
| | A4 | 13.9 (13) | 19.6 (10) | 0.0 (0) | 27.2 (3) | 24.4 (11) | 57.1 (4) |
| **Percussion Marks** | A3 | 3.9 (2) | 0.0 (0) | 0.0 (0) | 0.0 (0) | 30.1 (16) | 0.0 (0) |
| | A4 | 2.2 (2) | 0.0 (0) | 0.0 (0) | 0.0 (0) | 11.1 (5) | 0.0 (0) |
| **Total NISP** | A3 | 51 | 20 | 14 | 13 | 53 | 6 |
| | A4 | 93 | 51 | 4 | 11 | 45 | 7 |
